# Isolated agenesis of the left pulmonary artery: A case report

**DOI:** 10.7196/AJTCCM.2021.v27i3.105

**Published:** 2021-10-04

**Authors:** M J Mpe, W van Aswegen

**Affiliations:** Division of Pulmonology, Department of Medicine, Sefako Makgatho Health Sciences University, South Africa

**Keywords:** unilateral, agenesis, pulmonary artery

## Abstract

We present a case of a middle-aged patient followed up at the medical outpatient’s department routinely over two years for hypertensive heart disease and tobacco-induced chronic obstructive pulmonary disease. The patient was found to have an additional problem of congenital absence of the left main pulmonary artery.

## Case

A 55-year-old truck driver was referred to our respiratory clinic for progressively worsening breathlessness and easy fatigability. He was known to have hypertensive heart disease for some years and had remained symptomatic despite treatment, which included diuretics, calcium channel blockers and angiotensin converting enzyme (ACE) inhibitors. The diagnosis of hypertensive heart disease was presumptive as no echocardiogram had been done at the time. He had persisting general body swelling and a 3-pillow orthopneoa. This had been ongoing for ~2 years. There was no associated cough, wheeze, chest tightness or pain. He gave no history of haemoptysis or recurrent chest infections. He was a current smoker with a 30-packs a year history. Alcohol intake was within recommended limits.

Clinically, he was comfortable at rest. He had central cyanosis, was plethoric and had bilateral pitting oedema up to his knees. There was no digital clubbing. There was jugular venous elevation to the angle of the jaw and a pulsatile hepatomegaly 4 cm below the costal margin. Lung examination was only remarkable for scattered inspiratory crackles. Cardiac auscultation revealed slight accentuation of the pulmonary component of the second heart sound and there were no murmurs. The apex was impalpable.

Spirometry confirmed chronic obstructive pulmonary disease (COPD) of moderate severity (forced expiratory volume in 1 second/forced vital capacity (FEV_1_ /FVC) 0.52 and FEV_1_ 1.66 (52%)). Arterial blood gas (ABG) analysis on breathing ambient air showed a PaO_2_ of 5.25 kPa and a PaCO_2_ of 5.55 kPa. The ABG on supplemental oxygen via nasal prongs at 3 L per minute showed a PaO_2_ of 8.28 kPa and PaCO_2_ of 5.38 kPa.

A subsequent echocardiogram confirmed severe pulmonary hypertension, a dilated right ventricle and severe tricuspid regurgitation. His left ventricular ejection fraction was normal at 73% with no suggestion of diastolic dysfunction. There were no other cardiac lesions. Apart from a comment on the pulmonary artery pressures, no mention was made regarding the status of the pulmonary arteries (PAs) themselves.

**Fig. 1:**
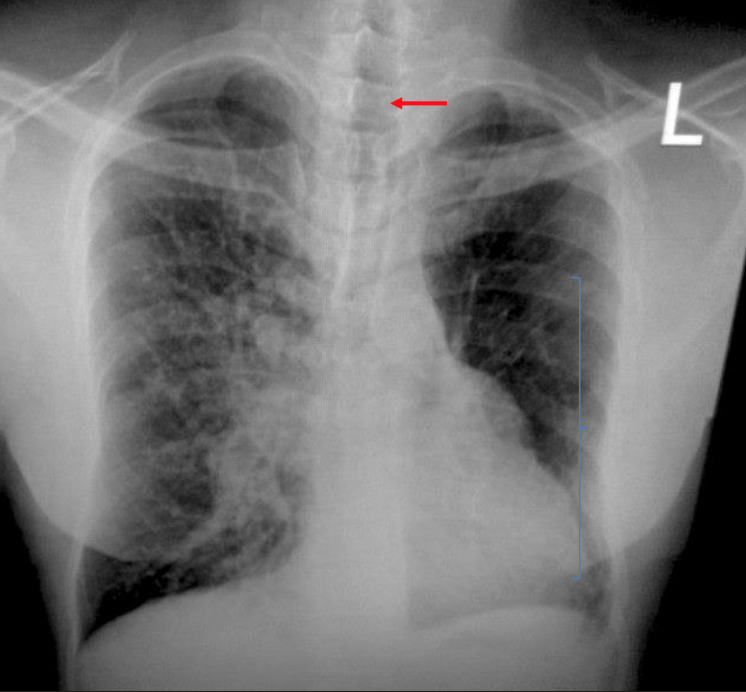
Chest X-ray showing reduction in the size of the left hemithorax, plethora of the right lung and leftward shift of the mediastinum.

His chest radiograph (Fig. 1) shows reduction in the size of the left hemithorax, plethora of the right lung and leftward shift of the mediastinum.

**Fig. 2:**
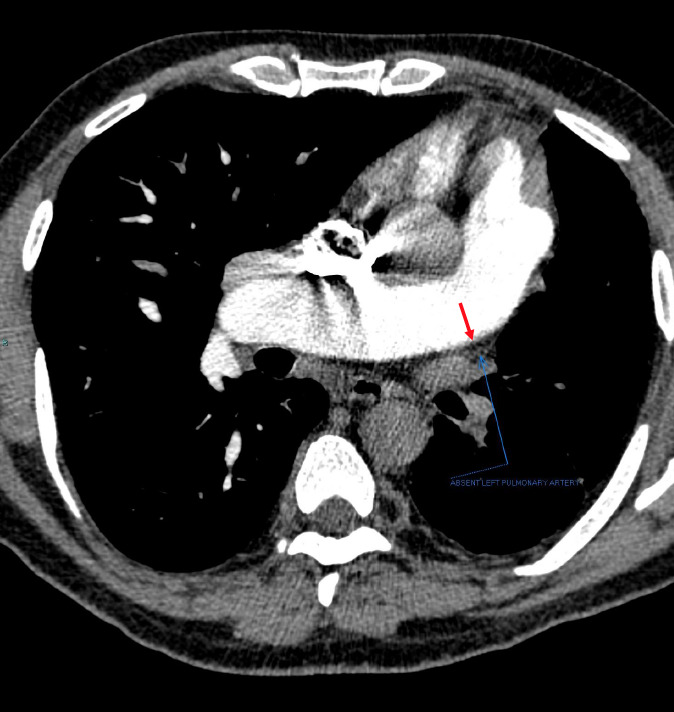
Absent left pulmonary artery. Clear fat plane at site of normal origin of the left pulmonary artery.

**Fig. 3:**
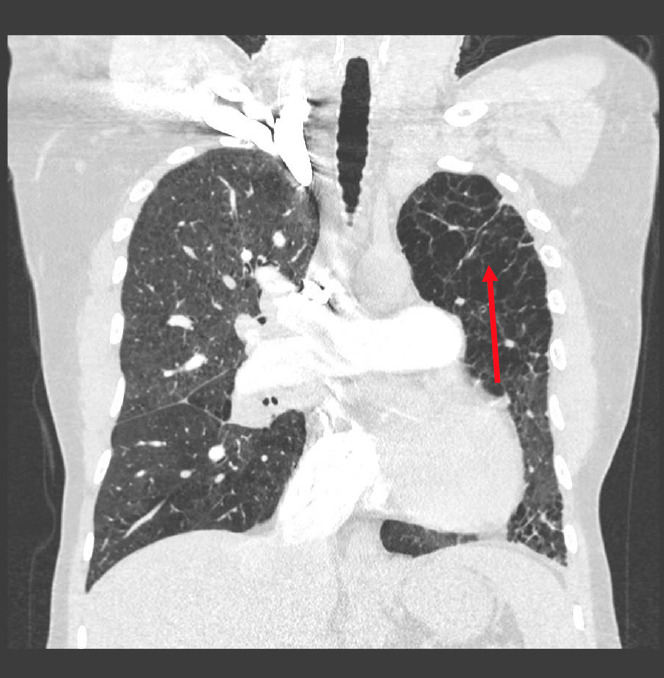
Hypoplastic and oligemic left lung.

**Fig. 4:**
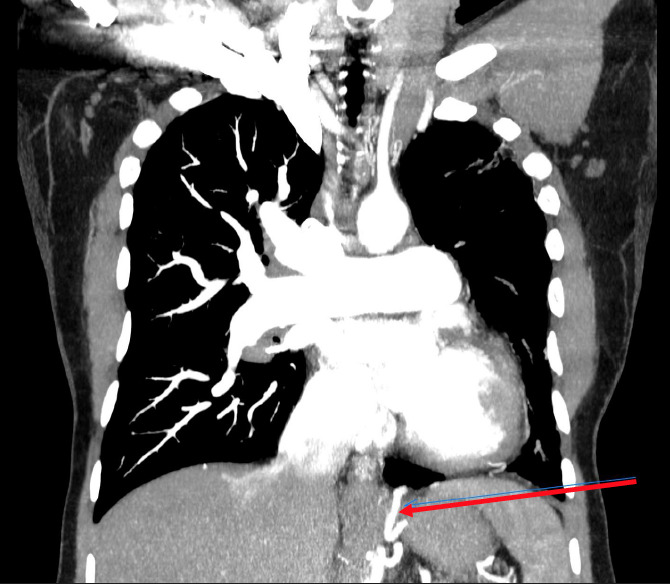
Collateral supply from the coeliac trunk.

**Fig. 5:**
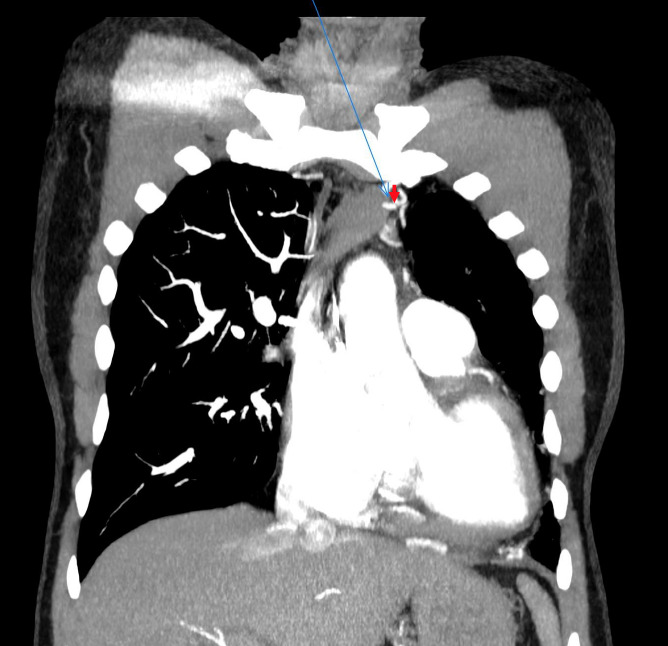
Collateral supply from the left subclavian artery.

Cuts from his computed tomography pulmonary angiogram are shown in Fig. 2 - 5. The left PA is absent as evidenced by a clear fat plane that envelopes the site of normal origin of the left PA from the trunk (Fig. 2).

The left pulmonary veins are present but poorly opacified. The left lung is hypoplastic and oligemic with collateral supply from the left subclavian, the descending thoracic aorta and coeliac trunk (Figs. 3, 4 and 5). There is bilateral panlobular emphysema.

Our final impression was that of cor pulmonale secondary to unilateral agenesis of the left pulmonary artery and moderately severe COPD. There was no echocardiographic evidence of long-standing hypertension. He was deemed an inappropriate candidate for surgical intervention. We did not think it was appropriate to offer him longacting oral pulmonary vasodilators and only added long-term oxygen therapy to his treatment regimen.

## Discussion

Unilateral agenesis of the pulmonary artery (UAPA) is an uncommon condition. The exact prevalence remains obscure as most patients remain asymptomatic for long periods of time. The incidence is estimated at 1 in 200 000 young adults.^[1]^ There is no gender predilection. It can occur in isolation, but left PA agenesis often occurs in association with other cardiac abnormalities such as tetralogy of Fallot, septal defects or patent ductus arteriosus.^[2]^ Isolated agenesis involves the right PA in two-thirds of cases.^[2,3]^

The anomaly results from involution of the proximal portion of the sixth aortic arch during embryogenesis, causing absence of the proximal PA on the affected side. The PA trunk is normal. The distal intrapulmonary branches usually remain intact and receive blood supply from systemic collaterals. Several variants of collateral supply have been described: bronchial, intercostal, internal mammary, sub-diaphragmatic, subclavian and coronaries.^[4,5]^

The clinical presentations are variable. Right PA agenesis can remain silent for many years and even throughout the patient’s life.^[1]^ The commonly described symptoms have been any of the following, either singly or in combination: recurrent pulmonary infections, exercise intolerance, haemoptysis and chest pain.^[1-3,6,7]^ Clinical pulmonary hypertension (PH) has been observed in a quarter of the cases and is thought to be an important determinant of survival.^[1,3,5]^

PH is thought to be secondary to increased blood flow through the existing PA, resulting from diversion of flow from the absent vessel. This leads to shear stress on the endothelium, release of vasoconstrictor substances (e.g. endothelin), chronic vasoconstriction, remodeling and increased resistance of the pulmonary vasculature.^[2]^ The recurrent chest infections are thought to be multifactorial in origin; lack of arterial blood-flow leading to poor delivery of inflammatory cells and impairment of muco-cilliary function, and alveolar hypocarbia which causes broncho-constriction and mucus trapping.^[1]^ Haemoptysis is thought to arise from the hypertrophied bronchial collaterals, peripheral arteriovenous fistulae ipsilateral to the absent PA or rupture of chronically hyper-perfused vessels on the contralateral side.^[8]^

There is no consensus regarding treatment. In adults, therapy is tailored to the patient’s clinical presentation. Vaccines to reduce the frequency of respiratory tract infections are likely to be helpful. Lobectomy or pneumonectomy are options available for recurrent haemoptysis or intractable pulmonary sepsis whiles surgical re-vascularisation can be considered for PH.^[2,3,9]^ Selective embolisation for massive haemorrhage from collaterals is an option in surgically unsuitable cases.^[7]^ Pharmacological treatment of pulmonary hypertension is recommended for those unable to undergo surgical re-vascularisation or fail to improve post surgery.^[10]^ Heart-lung transplantation has been suggested as a treatment option to improve oxygenation.^[9]^

The overall mortality rate from UAPA is ~7%, with common causes of death including right heart failure, respiratory failure and massive haemoptysis.^[1,3,7]^ Regular follow-up with echocardiography for early detection of PH is recommended.^[7]^

## References

[1] Ten Harkel AD, Blom NA, Ottenkamp J (2002). Isolated unilateral absence of a pulmonary artery: A case report and review of the literature.. Chest.

[2] Kruzliak P, Syamasundar RP, Novak M (2013). Unilateral absence of pulmonary artery: Pathophysiology, symptoms, diagnosis, and current treatment.. Arch Cardiovasc Dis.

[3] Hiroshi K, Tomoko H, Kenichi M (2010). Age-related clinical characteristics of isolated congenital unilateral absence of a pulmonary artery.. Paediatr Cardiol.

[4] Bockeria LA, Makhachev OA, Khiriev TKH, Abramyan MA (2011). Congenital isolated unilateral absence of pulmonary artery and variants of collateral blood supply of the ipsilateral lung.. Interact Cardiovasc Thorac Surg.

[5] De Dominicis F, Leborgne L, Raymond A, Berna P (2011). Right pulmonary artery agenesis and coronary-to-bronchial artery aneurysm.. Interact Cardiovasc Thorac Surg.

[6] De Mello Junior WT, Coutinho N JR, Santos M (2008). Isolated absence of the right pulmonary artery as a cause of massive hemoptysis.. Interact Cardiovasc Thorac Surg.

[7] Steiropoulos P, Archontogeorgis K, Tzouvelekis A (2013). Unilateral pulmonary artery agenesis: A case series.. HIPPOKRATIA.

[8] Launer BA, Serva S, Weyant M (2019). Surgical treatment of pulmonary artery agenesis.. Chest.

[9] Johnson TRC, Thieme SF, Deutsch MA (2009). Unilateral pulmonary artery agenesis. Non-invasive diagnosis with dual source computed tomography.. Circulation.

[10] Steiropoulos P, Trakada G, Bouros D (2008). Current pharmacological treatment for pulmonary arterial hypertension.. Curr Clin Pharmacol.

